# Inflammasome, clonal stability and the thrombotic circuit in myeloproliferative neoplasms: an antecedent framework

**DOI:** 10.1007/s10238-026-02343-9

**Published:** 2026-10-15

**Authors:** Alessandro Lucchesi, Mariasanta Napolitano

**Affiliations:** 1 Hematology Unit, IRCCS Istituto Romagnolo per lo Studio dei Tumori (IRST) “Dino Amadori”, Meldola, Italy; 2 Department of Health Promotion, Mother and Child Care, Internal Medicine and Medical Specialities (PROMISE), Internal Medicine and Medical Specialties, University of Palermo, Palermo, Italy; 3 Hematology and Rare Diseases Unit, Hospital V. Cervello, Palermo, Italy

## Abstract

El-Sehrawy and colleagues recently proposed a model of clonal thrombo-inflammation in myeloproliferative neoplasms organised around the inflammasome, clonal stability and a multi-compartmental thrombotic niche. Each of these elements was addressed in two reviews published by our group in 2021, neither of which is cited. The first set out the pathogenetic role of the NLRP3 and AIM2 inflammasomes and their link to hypercoagulable states; the second formalised the vascular consequence as the Circulating Wound, a self-sustaining lesion in which tissue-factor-rich clonal platelets, thrombin generation, protease-activated receptor signalling and TGF-β crosstalk reinforce one another. This coagulative arm is absent from the more recent model. We summarise experimental findings from 53 therapy-naive patients that support the earlier formulation, and set out where the two accounts diverge and why the difference bears on the therapeutic reading.

Dear Editor,

We read with interest the review by El-Sehrawy and colleagues, published on 28 June 2026 [1], which proposes an integrative model of clonal thrombo-inflammation in myeloproliferative neoplasms (MPN) organised around the inflammasome, clonal stability, and a multi-compartmental thrombotic niche. Each of the three elements named in their title, together with the departure from the mechanistic model of clonal evolution announced in their opening, was addressed in two companion reviews published by our group in 2021, neither of which appears among their references. We raise this not for attribution alone, but because those earlier formulations differ from theirs in a respect that bears directly on the therapeutic reading.

The first [2], published on 8 January 2021, addressed the genetics and pathogenetic role of inflammasomes in Philadelphia-negative MPN. Building on Hasselbalch’s paradigm of MPN as inflammatory disease [3], it proposed replacing the purely mechanistic model of clonal evolution with one in which chronic inflammation and immune dysregulation are constitutive, and identified NLRP3 and AIM2 as the paired sensors governing cytokine release, atherosclerotic acceleration and fibrotic progression. A dedicated section addressed inflammasome activation and hypercoagulable states: constitutive NLRP3 expression in platelets requiring neither priming nor upstream activation, tissue factor released by macrophages undergoing pyroptosis, caspase-1-mediated shedding of thrombo-inflammatory extracellular vesicles, endothelial pyroptosis, and BTK as a modulator of platelet NLRP3. A further section proposed BET inhibition (then CPI-0610, now pelabresib), IRAK4 inhibition and BTK inhibition as strategies directed at the inflammatory circuit rather than at the clone.

The second [4], published on 20 October 2021, formalised the vascular consequence of that biology as the *Circulating Wound*: a self-sustaining lesion in which tissue-factor-rich clonal platelets, thrombin generation, protease-activated receptor signalling and TGF-β crosstalk reinforce one another in a vicious circle linking coagulation, inflammation and fibrotic progression within a single circuit. This coagulative arm is the respect in which the two accounts diverge. The thrombotic niche of El-Sehrawy et al. is assembled from cytokines, adhesion molecules and neutrophil extracellular traps, and stops short of the tissue factor, thrombin and PAR axis through which clonal platelets convert inflammation into fibrin.

Their model partitions the pathological space into compartments and characterises their interaction, which motivates disruption of individual effectors. The *Circulating Wound* is a circuit rather than a map: it emphasises auto-perpetuation and reads clonal expansion as both consequence and stabiliser of the inflamed microenvironment rather than solely as its cause, which motivates instead restoring the physiological silencers that would return it to quiescence. On the selective function of inflammation the two accounts agree, El-Sehrawy et al. describing systemic inflammation as *“a Darwinian selection pressure that stabilizes clonal dominance”* [1].

This is not only a conceptual claim. In 53 therapy-naïve patients with myeloproliferative neoplasms we interrogated the circuit directly, combining rotational thromboelastometry with a 30-gene sequencing panel and cytofluorimetric quantification of platelet fibrinogen receptors [5]. Coagulation behaviour segregated by disease: essential thrombocythaemia preserved the balance of both pathways, polycythaemia vera showed prolonged clot formation times (EXTEM 114 s, *p* = 0.008; INTEM 82 s, *p* = 0.027), and primary myelofibrosis shifted towards the extrinsic route. Within each of the latter two, clot formation accelerated as the disease expanded: in myelofibrosis the extrinsic time shortened as leukocyte and platelet counts rose, in polycythaemia vera the intrinsic time did so, and mutations in DNA-methylation regulators tracked with further changes in clotting parameters. Coagulation efficiency therefore scales with proliferative burden along a route that differs by disease, and in the most fibrotic subtype that route is the tissue-factor-triggered one. This is what the Circulating Wound model predicts, and it is the arm a compartment map of cytokines and neutrophil extracellular traps cannot express.

The principle is not confined to MPN. In a Perspective now in preparation we extend it across paroxysmal nocturnal haemoglobinuria, AL amyloidosis, systemic mastocytosis, immune thrombocytopenia and MPN, proposing that thrombo-inflammation operates as an archaic co-evolved module of coagulation and innate immunity [6] whose chronic engagement becomes a clonal selector, in the lineage of the perpetual cycle between clonal haematopoiesis and inflammation [7]. Its genealogy passes through the two reviews cited here. The recurrence of this architecture across groups signals a maturing paradigm, whose standing will be strengthened by an accurate genealogical record.

## Data Availability

No new data were generated for this Correspondence. The experimental findings summarised here were previously presented in abstract form at the 2022 American Society of Hematology Annual Meeting (Blood 2022;140(Supplement 1):12160-12161, doi 10.1182/blood-2022-171095), which is publicly available. The complete dataset will be made available with the full study, currently in preparation.

## Declarations

### Statement on the use of AI tools

During the preparation of this work, the authors used Claude (Anthropic) to assist with text editing, structural reorganization, and language refinement. The authors thoroughly reviewed, verified, and edited all outputs for accuracy and consistency. The authors assume full accountability for the content and integrity of the final publication.
